# The Therapeutic Potential of Berry-Derived and Berry-Based Interventions in Inflammatory Bowel Disease: A Comprehensive Systematic Review of Preclinical and Clinical Evidence

**DOI:** 10.3390/nu18193280

**Published:** 2026-10-06

**Authors:** Lucia Carmela Passacatini, Rosamaria Caminiti, Valeria Mazza, Saverio Nucera, Stefania Proietti, Francesca Oppedisano, Jessica Maiuolo, Roberta Macrì, Maria Serra, Valentina Malafoglia, Federica Marrelli, Andrea Soluri, Myriam Carpenito, Carlo Tomino, Vincenzo Mollace, Sara Ilari, Carolina Muscoli

**Affiliations:** 1 Pain Physiology and Pharmacology, IRCCS San Raffaele Roma, 00166 Rome, Italy; carmela.passacatini@sanraffaele.it (L.C.P.);; 2 Department of Health Sciences, Institute of Research for Food Safety and Health (IRC-FSH), University “Magna Graecia” of Catanzaro, 88100 Catanzaro, Italyoppedisanof@libero.it (F.O.);; 3 Agea, Coordinating Body, 00185 Rome, Italy; 4 Department of Health Sciences, University “Magna Graecia” of Catanzaro, 88100 Catanzaro, Italy; federica.marrelli@unicz.it; 5 Department of Medical and Surgical Sciences, University “Magna Graecia” of Catanzaro, 88100 Catanzaro, Italy; andrea.soluri@unicz.it; 6 Cardiology Unit, Fondazione Policlinico Universitario Campus Bio-Medico, 00128 Rome, Italy; 7 Research Unit of Cardiovascular Science, Department of Medicine and Surgery, Università Campus Biomedico di Roma, 00128 Rome, Italy; 8 San Raffaele Sulmona, Viale dell’Agricoltura, 67039 Sulmona, Italy; 9 Department of Human Sciences and Quality of Life Promotion, San Raffaele University, 00166 Rome, Italy

**Keywords:** berries, inflammatory bowel diseases, berry-based interventions, dietary, chronic inflammation

## Abstract

Introduction: Berry-derived and berry-based interventions have gained increasing attention for their potential health benefits. Berries are rich in diverse bioactive compounds, including polyphenols and anthocyanins, which are associated with a broad range of biological activities, particularly antioxidant and anti-inflammatory effects. Over the last decade, numerous studies have focused on berries, particularly those from the Ericaceae family (blueberry, cranberry and bilberry), the Solanaceae family (goji berry, *Solanum nigrum* berries) and the Elaeocarpaceae family (maqui berry). Berries are considered among the best dietary sources of bioactive compounds (BACs). Among chronic gastrointestinal disorders, inflammatory bowel disease (IBD) mainly includes Crohn’s disease (CD) and ulcerative colitis (UC), both chronic inflammatory conditions of the gastrointestinal tract. The multifactorial nature of IBD significantly impairs quality of life and makes disease management particularly challenging, increasing interest in complementary approaches, in addition to pharmacological treatments, such as phytotherapeutic strategies based on plant-derived bioactive compounds and, in particular, berry-derived and berry-based interventions. Methods: A systematic literature search was conducted using PubMed, Web of Science, and Scopus databases. Specific inclusion and exclusion criteria were applied to identify relevant articles published up to July 2026. After the removal of duplicates and a thorough screening of titles, abstracts, and full texts, a total of 19 studies were included, comprising 16 studies conducted on animal models and 3 clinical studies involving patients. The 3 clinical studies corresponded to 5 publications and involved a total of 68 unique participants, as two publications were secondary analyses of the same patient cohorts. These investigations evaluated the possible effects of berry-derived and berry-based interventions in both experimental models and clinical settings. Results: Preclinical studies suggested potential protective effects against intestinal inflammation by reducing pro-inflammatory cytokines, improving epithelial barrier integrity, and restoring gut microbiota composition. The included clinical studies reported improvements in disease activity, fecal calprotectin levels, quality of life, and remission rates in patients with ulcerative colitis. Nevertheless, the available clinical evidence remains limited and heterogeneous, preventing definitive conclusions regarding clinical efficacy. Conclusions: The current clinical data should therefore be considered preliminary. Larger and more rigorous randomized controlled trials are needed to determine whether the biological effects observed in preclinical models translate into clinically relevant benefits in patients with IBD. Overall, preclinical evidence complements clinical findings by providing mechanistic insights that help contextualize and interpret outcomes observed in human studies.

## 1. Introduction

### 1.1. Exploring Berry-Derived and Berry-Based Interventions: Classification and Properties

Polyphenols, a class of naturally occurring chemical compounds widely distributed in the plant world, have attracted significant attention for their potential health benefits. They are characterized by the presence of multiple phenolic rings and hydroxyl groups, which give them various biological properties [1]. Polyphenols are classified into several subgroups, mainly including flavonoids (such as flavones, flavanols, flavanones, flavonols, anthocyanins, and isoflavones) and non-flavonoids: phenolic acids (such as hydroxybenzoic acids and hydroxycinnamic acids), stilbenes, lignans, and tannins [2] (Figure 1).

The most important food sources of polyphenols include coffee, tea, fruits and vegetables, cocoa, soy products, nuts, olive oil, red wine, cereals, and whole grains.

Berries have gained significant scientific interest because they are among the richest dietary sources of bioactive compounds (BACs). Belonging to several botanical families, including blueberries, bilberries, cranberries, goji berries, maqui berries, blackcurrants, and grapes, they are widely consumed and commonly incorporated into functional foods. Their main bioactive constituents include phenolic compounds that exhibit strong antioxidant, anti-inflammatory, immunomodulatory, and gut microbiota-modulating properties. In particular, anthocyanins and proanthocyanidins can target key mechanisms involved in IBD, such as oxidative stress, intestinal barrier dysfunction, immune dysregulation, and dysbiosis. Owing to their complex phytochemical profile and multitarget mode of action, berries are considered promising dietary adjuncts for the prevention and management of IBD.

Polyphenols reduce oxidative stress by scavenging reactive oxygen and nitrogen species (ROS and RNS) [3], inhibiting ROS-generating enzymes, and enhancing endogenous antioxidant defenses such as SOD, catalase, and glutathione peroxidase (GPx) [4,5,6]. In addition, polyphenols promote beneficial gut microbiota composition and the production of bioactive microbial metabolites that support intestinal barrier integrity and immune homeostasis [7,8,9,10,11]. Polyphenols also modulate immune responses by promoting regulatory T-cell activity and reducing pro-inflammatory signaling pathways, mechanisms that are particularly relevant in inflammatory bowel diseases. Experimental studies have shown that epigallocatechin-3-gallate (EGCG) increases Treg-associated markers, including Foxp3 and IL-10, supporting its anti-inflammatory potential [12,13,14].

### 1.2. Inflammatory Bowel Diseases and Therapy

Crohn’s disease (CD) and ulcerative colitis (UC), the two main forms of inflammatory bowel disease (IBD), are both classified as IMIDs [15]. Both disorders have a complex and multifactorial pathogenesis without a clearly identified primary cause, and diagnosis is mainly based on exclusion criteria. CD can affect any region of the gastrointestinal tract and involves the entire intestinal wall, whereas UC is limited to the mucosa and submucosa of the colon [16,17]. Globally, approximately 6.8 million people are affected by IBD, with the highest prevalence in North America and the United Kingdom [18]. Common symptoms include chronic abdominal pain, cramping during bowel movements, and the presence of blood, pus, or mucus in the stool. Beyond intestinal manifestations, persistent pain represents one of the most disabling symptoms, substantially affecting patients’ quality of life, psychological well-being, sleep quality, and social functioning, even during periods of clinical remission [14,19,20].The therapeutic goal in IBD is to induce and maintain remission and prevent disease progression. Current pharmacological treatments include 5-aminosalicylates (5-ASA), corticosteroids, immunosuppressants, biologics, and Janus kinase (JAK) inhibitors. The 5-ASA drugs are mainly used for mild-to-moderate UC, while corticosteroids are effective for inducing remission during acute flares but are unsuitable for long-term use due to adverse effects [15,21]. Immunosuppressants such as azathioprine, mercaptopurine, and methotrexate are used as maintenance therapies. Biological agents, including anti-TNFα antibodies and anti-integrin therapies, are essential for patients with moderate-to-severe or refractory disease [22,23,24,25].

JAK inhibitors such as tofacitinib are used to treat moderate or severe UC by blocking intracellular signaling pathways that promote inflammation [26]. JAKs are intracellular tyrosine kinases that mediate cytokine signaling through signal transducer and activator of transcription (STAT) proteins; dysregulation of this pathway contributes to inflammatory responses in IBD [27,28,29]. In addition to pharmacological therapies, growing attention has been given to dietary bioactive compounds.

In parallel with established pharmacological treatments, increasing attention has been directed toward dietary bioactive compounds, particularly polyphenols. These phytochemicals, including flavonoids and anthocyanins, can regulate inflammation through different pathways, including protein kinase C and mitogen-activated protein kinase. By inhibiting these enzymes, these phytochemicals interfere with the ability of transcription factors like NF-κB to bind to DNA [30]. The health benefits of these compounds are primarily associated with the antioxidant and anti-inflammatory properties of polyphenols found in fruits and vegetables [31,32], which may play an important role in immunomodulation and contribute to the prevention and management of various chronic diseases. (Figure 1).

### 1.3. Interplay Between Polyphenols and the Immune System in IBD

Beyond their antioxidant activity, polyphenols have emerged as important modulators of immune responses and gut homeostasis. Their beneficial effects are particularly relevant in IBD, where chronic inflammation results from a complex interplay among the immune system, the intestinal barrier, and the gut microbiota. By modulating inflammatory signaling pathways and immune cell function, polyphenols have also been proposed as potential adjuvants to enhance the efficacy of conventional pharmacological therapies in the management of IBD.

Proper immune system function and the tightly regulated activation of immune cells are essential for maintaining intestinal immune homeostasis. Dysregulation of these processes contributes to the development and progression of immune-mediated inflammatory diseases, including IBD [20,33,34].

IBD pathogenesis involves an imbalance between innate and adaptive immune responses. Patients with active Crohn’s disease show reduced natural killer (NK) cell expression compared with healthy controls, suggesting impaired innate immune activity during disease progression. Moreover, interactions among epithelial cells, dendritic cells, and T cells contribute to the regulation of intestinal inflammation and disease development [14,35,36,37].

In this context, gut microbiota plays a pivotal role in shaping host immune responses through the production of bioactive metabolites, including short-chain fatty acids, tryptophan metabolites, and bile acid derivatives. These metabolites contribute to intestinal and systemic immune homeostasis by promoting regulatory immune pathways and suppressing excessive inflammatory responses. Alterations in microbial composition and metabolite production may therefore contribute to IBD pathogenesis [38].

Furthermore, dietary polyphenols can interact with the gut microbiota, influencing microbial composition and generating bioactive metabolites that may contribute to the regulation of intestinal inflammation and immune responses [19]. Given these promising biological properties, this systematic review aims to critically summarize the available preclinical and clinical evidence regarding the therapeutic potential of berry-derived and berry-based interventions in IBD, with particular emphasis on their effects on intestinal inflammation, immune regulation, oxidative stress, gut microbiota, and clinical outcomes.

## 2. Materials and Methods

This systematic review includes published data from preclinical and clinical studies investigating berry-derived and berry-based interventions in inflammatory bowel disease (IBD), with particular attention to their phytochemical and polyphenol-related properties. The studies included in the review were retrieved from the PubMed, Web of Science, and Scopus databases in accordance with the PRISMA (Preferred Reporting Items for Systematic Reviews and Meta-Analyses) statement and following the PICOs framework (Population Intervention Comparison Outcome Study design) (Table 1 and Table 2).

The search included studies published up to July 2026. All papers in English were evaluated. The reference list of all retrieved articles was also reviewed to identify other eligible studies that were not indexed by the above-mentioned databases.

The keywords used for the research of the articles were on PubMed: (“Berries” [mh] OR Berries OR “Fruit” [mh] OR “Plant Extracts” [mh]) AND (“Inflammatory Bowel Diseases” [mh] OR “Colitis, Ulcerative” [mh] OR “Crohn Disease” [mh] OR “Inflammatory Bowel Disease”) AND (Polyphenols OR “Polyphenols” [mh] OR “Flavonoids” [mh] OR “Anthocyanins” [mh]).

This PubMed strategy was adapted for Web of Science, as follows.

Web of Science: (TS = (berry OR berries OR “berries polyphenols*” OR “fruit extract*” OR “plant extract*”) AND TS = (“inflammatory bowel disease*” OR ibd OR “crohn disease” OR “ulcerative colitis”) AND TS = (polyphenol* OR flavonoid* OR anthocyanin* OR “bioactive compound*”)

Scopus: (TITLE-ABS-KEY (berry OR berries OR “fruit extract*” OR “plant extract*”) AND TITLE-ABS-KEY (“inflammatory bowel disease*” OR ibd OR “crohn disease” OR “ulcerative colitis”) AND TITLE-ABS-KEY (polyphenol* OR flavonoid* OR anthocyanin* OR “bioactive compound*”).

Only articles published in English were considered. The last search was conducted in July 2026.

This systematic review was registered on PROSPERO in accordance with its requirements, with separate registrations submitted for the preclinical (CRD420251230910) and clinical (CRD420251230822) systematic reviews. The two registrations correspond to the preclinical and clinical components of the present systematic review, respectively. No substantive deviations from the registered protocols occurred.

### 2.1. Literature Search and Study Selection

All records retrieved from the database searches were collected and organized manually. In the initial screening phase, duplicates were identified and removed. Subsequently, document types not relevant to the study were excluded, including reviews, literature reviews, narrative reviews, book chapters, letters, documents, and books, selecting exclusively original research articles. Two independent reviewers (L.C.P. and R.C.) screened all titles and abstracts for eligibility. Full texts of potentially relevant articles were retrieved and assessed independently by the same reviewers. Any disagreements were resolved through discussion; when consensus could not be reached, a third reviewer (S.N.) was consulted.

Data from the studies included were extracted by one author (L.C.P.) and independently verified by others (S.I., S.N., V.M., R.C.). Extracted information included: first author and year of publication, country, study design, animal model or patient population (sample size, age, sex, disease type), type of berry or berry-derived product and formulation, dose and route of administration, duration of treatment, main outcomes assessed, and key findings. This process, conducted entirely through independent manual assessment by multiple reviewers, ensured a rigorous and reproducible selection of relevant literature, minimizing study selection bias and allowing for a comprehensive identification of the available scientific evidence on the research topic.

#### 2.1.1. Data Synthesis

Owing to the substantial methodological and clinical heterogeneity among the included studies in terms of experimental models or patient populations, intervention formulations, doses, routes of administration, treatment duration, comparators, and outcome measures, a quantitative meta-analysis was not considered appropriate. Therefore, the findings were synthesized narratively. Preclinical and clinical evidence was analyzed separately, and studies were compared according to disease or experimental model, intervention characteristics, dose and duration, comparator, and main outcome domains. Where available, quantitative findings and measures of statistical uncertainty were considered together with the direction and statistical significance of the reported effects. Multiple publications arising from the same patient cohort were treated as related reports of a single study and were not counted as independent studies or independent participant populations.

#### 2.1.2. Eligibility Criteria

Preclinical Studies

Inclusion criteria:

- Original in vivo studies using experimental models of IBD (e.g., DSS, TNBS, IL-10^−^/^−^ colitis).
- Studies investigating berry-derived or berry-based interventions, including whole-fruit preparations, extracts, juices, powders, fermented products, oils, polysaccharide-rich preparations, lipid-derived formulations, and phytochemically characterized fractions.
- Studies reporting at least one quantitative outcome related to inflammation, oxidative stress, intestinal barrier function, or gut microbiota.
- Studies published in peer-reviewed journals in English.

Exclusion criteria:

- In vitro studies, in silico studies and ex vivo only experiments.
- Studies not using an IBD-related model.
- Studies without quantitative outcome measures.
- Reviews, editorials, letters, and conference abstracts.

Clinical studies:

Inclusion criteria:

- Clinical trials (randomized or non-randomized), pilot studies, or prospective interventional studies.
- Adult patients diagnosed with ulcerative colitis or Crohn’s disease.
- Interventions based on berry-derived or berry-based products, including whole-fruit preparations, extracts, juices, powders, and standardized phytochemical formulations.
- Studies reporting at least one clinical, biochemical, or microbiological outcome (e.g., disease activity scores, fecal calprotectin, cytokines, microbiota composition, quality of life).

Exclusion criteria:

- Observational studies without intervention.
- Case reports.
- Studies not reporting measurable outcomes related to disease activity.
- Non-English publications.

#### 2.1.3. Quality Assessment and Risk of Bias Assessment

The methodological quality of the included studies was assessed using validated risk-of-bias tools appropriate to each study design. Randomized controlled trials (RCTs) were assessed using the Cochrane Risk of Bias 2 (RoB 2) tool, non-randomized studies of interventions were assessed using the Risk Of Bias In Non-randomized Studies of Interventions (ROBINS-I) tool, and preclinical animal studies were assessed using the SYRCLE Risk of Bias Tool. Assessments were conducted across all relevant domains of each instrument to identify potential sources of bias that could affect the internal validity of the included evidence. The results were summarized in risk-of-bias tables and presented as the distribution of risk-of-bias judgments across the assessed domains.

For randomized controlled trials, the RoB 2 tool was used to assess five domains: (1) bias arising from the randomization process; (2) bias due to deviations from the intended interventions; (3) bias due to missing outcome data; (4) bias in measurement of the outcome; and (5) bias in selection of the reported result. Each domain was judged as having a low risk of bias, some concerns, or a high risk of bias, according to the RoB 2 framework.

For non-randomized studies of interventions, the ROBINS-I tool was used to assess seven domains: (1) bias due to confounding; (2) bias in selection of participants into the study; (3) bias in classification of interventions; (4) bias due to deviations from intended interventions; (5) bias due to missing data; (6) bias in measurement of outcomes; and (7) bias in selection of the reported result. Each domain was classified as having low, moderate, serious, or critical risk of bias, or as having no information, according to the ROBINS-I framework. For single-arm pre-post studies, the risk of bias assessment was performed within the ROBINS-I framework, with specific methodological considerations. In the absence of a concurrent control group, it is not possible to distinguish intervention-related effects from changes occurring independently of the intervention, including temporal fluctuations in disease activity, regression to the mean, concomitant treatments, and other time-varying factors. For this reason, the confounding domain was assessed with particular attention to the lack of a concurrent comparator, and the domains of selection of participants and deviations from intended interventions were also considered in light of this limitation. The overall risk of bias was determined according to the ROBINS-I framework and its domain-specific algorithms, and the observed within-study changes were interpreted cautiously as preliminary evidence rather than as definitive estimates of intervention effects. For preclinical animal studies, the SYRCLE Risk of Bias Tool was used to assess 10 methodological items: (1) sequence generation; (2) baseline characteristics; (3) allocation concealment; (4) random housing; (5) blinding of caregivers and/or investigators; (6) random outcome assessment; (7) blinding of outcome assessors; (8) incomplete outcome data; (9) selective outcome reporting; and (10) other sources of bias. Each item was classified as having a low, high, or unclear risk of bias according to the signaling questions of the SYRCLE tool. When sufficient information was available to support a methodological judgment, the corresponding risk of bias rating was assigned. When insufficient information was available to determine whether a methodological procedure had been adequately performed, the domain was classified as having an unclear risk of bias rather than assuming a low risk of bias.

Risk-of-bias assessments were performed for each included study. The results were summarized in risk-of-bias tables, reporting the judgment assigned to each domain for each study and, where applicable, the overall risk-of-bias judgment according to the respective assessment tool.

## 3. Results

### 3.1. Data Collection

After a thorough screening process, 19 studies were included (16 preclinical and 3 clinical), corresponding to 21 publications, evaluating the effects of berry-derived and berry-based interventions in IBD patients and experimental animal models. The systematic review initially identified 919 records from the literature search (PubMed n = 667; Web of Science n = 103; Scopus n = 149).

After eliminating 175 duplicates, 744 records were screened. Of these, 270 records were excluded because they were reviews (n = 124), systematic reviews (n = 6), meta-analyses (n = 8), books or book chapters (n = 6), editorials or letters (n = 5), in vitro studies (n = 77), non-English articles (n = 10), or other records that were not relevant at title/abstract screening stage (n = 34). The remaining 474 reports were sought for retrieval; all 474 reports were assessed for eligibility. Of these, 453 reports were excluded because they did not meet the inclusion criteria, including studies that did not use an IBD-related model or did not report measurable outcomes related to disease activity (n = 114), observational studies without an intervention (n = 66), and studies that did not use berry-derived or berry-based interventions (n = 273).

Ultimately, 19 studies were included (16 preclinical and 3 clinical), corresponding to 21 publications (16 preclinical and 5 clinical). The clinical studies involved a total of 68 unique participants (13 in the Biedermann et al. 2013 [39] cohort, 34 in the Biedermann et al. 2024 trial [40], and 21 in the Eftekhar et al. 2026 [41] study), as two publications were secondary analyses of the same cohorts. The literature search and screening process is detailed in Figure 2.

### 3.2. Preclinical Evidence

Across the preclinical studies included in this systematic review, berry-derived and berry-based interventions were generally associated with beneficial effects in experimental models of inflammatory bowel disease (IBD), including dextran sodium sulfate (DSS)-, 2,4,6-trinitrobenzenesulfonic acid (TNBS)-, and IL-10^−^/^−^-induced colitis. The reported effects involved improvements in clinical, histological, inflammatory, oxidative, and microbiota-related outcomes associated with IBD pathophysiology.

Several studies investigating blueberry-derived preparations reported improvements in disease severity and inflammatory and oxidative stress parameters. Wang J. et al. (2024) [42] investigated the protective effects of blueberry extract on dextran sodium sulfate (DSS)-induced colitis, a mouse model of IBD. Mice treated with blueberry extract showed significant improvements compared to controls, including reduced body weight loss and less shortening of the colon length. Histological analysis revealed decreased colonic inflammation and tissue damage. These effects were accompanied by suppression of endoplasmic reticulum (ER) stress markers, including GRP78 and CHOP, and reduced caspase-3 activity, suggesting attenuation of ER stress-associated apoptosis in the colon [42].

Consistent with these findings, Osman et al. (2008) reported reductions in disease activity index (DAI), colonic myeloperoxidase (MPO) activity, and malondialdehyde (MDA) levels, following blueberry treatment [43]. In addition, blueberry treatment also reduced faecal Enterobacteriaceae counts, whereas changes in MCP-1 and GRO/CINC-1 did not reach statistical significance. Håkansson et al. (2009) similarly reported reduced disease activity, colonic malondialdehyde (MDA), serum interleukin-12 (IL-12) concentrations, and caecal Enterobacteriaceae abundance in animals receiving blueberry husks [44]. Interestingly, Enterobacteriaceae abundance was positively correlated with colitis severity, suggesting a possible relationship between microbiota alterations and disease severity.

In a TNBS-induced colitis model in rats, Pereira et al. (2017) showed that a blueberry anthocyanin-rich fraction (ARF) (about 10.28 ± 0.48 g/L, or 98.75 ± 5.71 mg/100g of fruit) improved recovery of body weight and reduced macroscopic colonic damage more effectively than 5-aminosalicylic acid (5-ASA) [45]. These effects were accompanied by reduced leukocyte infiltration, oxidative stress, and COX-2 and iNOS expression, suggesting a stronger anti-inflammatory and antioxidant profile than that of standard therapy. Overall, the findings suggest a potential protective effect of this anthocyanin mixture in reducing colitis severity and promoting faster recovery.

Similar protective effects were observed with Goji berry (wolfberry, *Lycium barbarum*) and its derived preparations. Dietary supplementation with whole Goji berry attenuated DSS-induced colitis, reduced disease activity index (DAI), colon shortening, and attenuated the reduction in mesenteric fat weight. In addition, wolfberry supplementation reduced plasma matrix metalloproteinase-3 (MMP-3) levels and tended to improve plasma interleukin-6 (IL-6). Transcriptomic and proteomic analyses further indicated downregulation of inflammation- and fibrosis-related genes and proteins, together with increased expression of proteins involved in epithelial protection and mucus barrier function [46]. Kang et al. (2017) [47] reported comparable findings, showing that Goji berry supplementation (1% Goji berry powder (*w*/*w*) for 4 weeks before colitis induction with 3% DSS in drinking water for 7 days, followed by a 7-day recovery period while maintaining the respective diets) attenuated body weight loss, diarrhea, fecal bleeding, DAI, and colon shortening, completely prevented the 30% mortality observed in DSS-treated control mice, and reduced histological damage, including mucosal injury, edema, crypt damage, and inflammatory cell infiltration. The protective effects were associated with reduced neutrophil infiltration and suppression of IL-6, COX-2, CXCL1, and MCP-1 expression. MMP-9 was also numerically reduced. Although numerical improvements were observed in tight-junction and adhesion molecule markers, these changes did not reach statistical significance [47]. In an IL-10-deficient model of spontaneous intestinal inflammation, Goji supplementation (1% Goji berry powder (*w*/*w*) for 10 weeks) reduced colonic histopathological damage and macrophage infiltration and improved epithelial integrity. These effects were accompanied by increased Muc1 expression and fecal IgA, reduced colonic Il17a and Tgfb1 expression, and marked increases in Bifidobacteriaceae/Bifidobacterium, Lachnospiraceae, Ruminococcaceae, and several butyrate-producing bacterial taxa. These microbiota changes were accompanied by an increase in the fecal butyryl-CoA:acetate CoA-transferase gene and increased fecal butyrate content, suggesting enhanced microbial butyrate production [48].

Probiotic-fermented Goji berry juice (20 mL/kg/day for 30 days) also improved DAI, body-weight loss, colon shortening, and histopathological damage in DSS-induced colitis. Both fermented and unfermented preparations reduced pro-inflammatory mediators and intestinal permeability and modified gut microbiota composition, while the fermented preparation exerted greater effects on selected inflammatory and oxidative stress markers [49].

In addition, Goji berry, Lycium barbarum lipid-derived nanoparticles (LBLNs), administered orally once daily at doses of 1 or 2 mg/kg of total flavonoids for 5 days in a mouse model of dextran sulfate sodium (DSS)-induced ulcerative colitis, accumulated in inflamed colonic tissue, suggesting their potential use as a targeted delivery strategy for berry-derived compounds to sites of intestinal inflammation [50].

Maqui berry (Aristotelia chilensis) water extract (MWE-H; administered orally at 50, 100, or 200 mg/kg in drinking water for 7 days) produced comparable effects in both DSS- and TNBS-induced models. This extract improved disease activity, colon shortening, and histopathological damage, while reducing MDA, NO, MPO, IL-1β, TNF-α, iNOS, and COX-2 and increasing GSH and occludin expression [51]. In a TNBS-induced Crohn’s disease mice model, maqui extract also reduced tissue damage, lipid peroxidation and pro-inflammatory cytokines (TNF-α, IL-6 and IL-1β), while enhancing antioxidant enzyme activity and IL-10 expression. These effects were associated with inhibition of NF-κB signaling and activation of Nrf2 mediated antioxidant pathways, suggesting coordinated antioxidant and anti-inflammatory mechanisms [52].

Grape-derived interventions likewise showed anti-inflammatory and antioxidant activity. Concentrated grape juice (G8000^TM^; 1% (1.18 g/kg/day) or 2% (2.36 g/kg/day), starting either 24 h or 7 days after colitis induction) reduced TNF-α and iNOS immunoexpression and, at the lower dose, COX-2 expression, while also reducing peripheral blood DNA damage [53]. In interleukin-10 knockout (IL-10^−^/^−^) mice, grape seed extract reduced clinical disease severity and myeloperoxidase activity and was associated with favorable changes in gut microbiota composition, including increased abundance of *Lactobacillus* and *Bacteroides* [54].

Beneficial effects were also reported for bilberry and cranberry-derived interventions. Bilberries reduced disease severity, inflammatory cytokine secretion, histological damage, and inflammation-induced apoptosis of colonic epithelial cells in acute and chronic DSS-induced colitis [55].

Cranberry supplementation reduced colonic inflammation and improved histopathological features, accompanied by changes in gut microbiota composition, including increased Lactobacillus and Bifidobacterium and decreased Sutterella and Bilophila abundance [56].

Fresh nightshade juice (FNS), obtained from the edible berries of Solanum nigrum, was also reported to improve survival, delay disease onset, reduce body weight loss and clinical symptoms, partially restore colon length, and preserve intestinal morphology in DSS-induced colitis [57].

The characteristics of the included studies are detailed in Table 3.

### 3.3. Clinical Evidence

Clinical evidence on berry-derived and berry-based interventions in inflammatory bowel disease (IBD) remains limited and heterogeneous, with most available data concerning ulcerative colitis (UC) and only very limited evidence in Crohn’s disease (CD).

The three clinical studies included a total of 68 unique participants: 13 patients with mild-to-moderate UC in the open-label pilot study by Biedermann et al. (2013) [39], 34 patients with moderate-to-severe UC in the randomized controlled trial by Biedermann et al. (2024) [40], and 21 patients with CD in the pilot study by Eftekhar et al. (2026) [41].

Biedermann et al. (2013) [39] conducted an open-label pilot study in 13 patients with mild-to-moderate UC receiving standardized anthocyanin-rich bilberry preparation. After the 6-week treatment period, 63.4% of patients achieved remission and 90.9% showed a clinical response. The total Mayo score decreased from a mean of 6.5 at baseline to 3.6 after treatment (*p* < 0.001), while fecal calprotectin decreased from 778 to 305 μg/g (*p* = 0.049). Improvements were also observed in endoscopic and histological disease activity. However, disease activity and fecal calprotectin increased again after discontinuation of bilberry intake. No serious adverse events were reported. Given the small sample size and uncontrolled design, these findings should be considered [39].

Roth et al. (2016) [58] subsequently performed a mechanistic analysis using samples obtained from the same patient cohort reported by Biedermann et al. (2013) [39]; the study was therefore considered a secondary report rather than an independent clinical study. Among patients who responded to the anthocyanin-rich bilberry intervention, treatment was associated with reduced mucosal TNF-α and IFN-γ expression and reduced activation of NF-κB, together with increased IL-10 and IL-22 levels and reduced circulating TNF-α and MCP-1. These findings provide mechanistic support for potential immunomodulatory effects of bilberry anthocyanins but do not constitute independent evidence of clinical efficacy [58].

In a subsequent multicenter, randomized, double-blind, placebo-controlled Phase IIa trial, Biedermann et al. (2024) [40] evaluated an anthocyanin-rich bilberry extract (ACRE) in patients with active moderate-to-severe UC. The primary clinical outcome did not demonstrate a statistically significant superiority of ACRE over placebo, with a clinical response observed in 9 of 18 patients receiving ACRE and 3 of 8 receiving placebo (*p* = 0.278). Nevertheless, fecal calprotectin decreased from 1049 ± 1139 to 557 ± 756 μg/g in the ACRE group, whereas no reduction was observed in the placebo group, resulting in a significant between-group difference (*p* = 0.035). Serious adverse events were rare. Thus, although the trial identified a biological signal related to intestinal inflammation, clinical efficacy was not established [40].

Zobrist et al. (2024) [59] performed a secondary microbiome analysis using participants from the same randomized controlled trial conducted by Biedermann et al. (2024) [40]. No significant overall changes in gut microbiota composition were observed. However, ACRE abolished the positive association observed at baseline between *Haemophilus parainfluenzae* abundance and fecal calprotectin that was observed at baseline, without significantly reducing the abundance of this microorganism. These findings suggest a possible interaction between the intervention, specific microbial features, and intestinal inflammation, although they should be interpreted as exploratory mechanistic evidence rather than as an independent clinical trial [59].

Evidence in Crohn’s disease is substantially more limited. Eftekhar et al. (2026) [41] conducted a pilot intervention study in 21 adults with CD who received 45 g/day of freeze-dried grape powder for 21 days. The primary outcome concerned changes in fecal microbiome profiles. No significant overall changes were observed in Harvey–Bradshaw Index (HBI), fecal myeloperoxidase (MPO), or hsCRP. Nevertheless, fecal MPO decreased in 61.9% of participants, while 80% showed either a reduction or no change in HBI. Modest and individualized changes in gut microbiota composition were also observed. These findings provide preliminary evidence of potential microbiome-related effects, whereas effects on clinical disease activity and inflammatory markers remain uncertain and require confirmation in larger controlled trials [41].

The characteristics of these studies are detailed in Table 4.

### 3.4. Risk of Bias Assessment

#### 3.4.1. Pre-Clinical Studies

Risk of bias was assessed in 16 animal studies using the SYRCLE Risk of Bias Tool. Overall, most domains were predominantly classified as unclear risk of bias, mainly due to insufficient reporting of methodological procedures. No domain was classified as high risk of bias.

Baseline characteristics represented the most consistently reported methodological aspect: 84% of studies were judged to have a low risk of bias and 16% an unclear risk. Sequence generation was classified as having a low risk in 28% of studies and an unclear risk in 72%. Allocation concealment, random housing, blinding, and random outcome assessment were judged to have an unclear risk of bias in all studies (100%), reflecting insufficient information on the methods used to implement these procedures. Blinding of outcome assessors was classified as unclear in 88% and a low risk of bias in 12%. Incomplete outcome data and other potential sources of bias were more frequently classified as having a low or unclear risk of bias, whereas selective outcome reporting was predominantly judged as unclear.

SYRCLE assessment indicated that the main limitation of the included animal studies was inadequate reporting of methodological procedures intended to minimize bias. Overall distribution of risk-of-bias assessments across the SYRCLE domains is illustrated in Figure 3.

#### 3.4.2. Clinical Studies

Three unique clinical studies were assessed for risk of bias using the appropriate Cochrane risk of bias tools. One randomized controlled trial was assessed using RoB 2, while two non-randomized single-arm studies were assessed using ROBINS-I. Among the two non-randomized single-arm studies, both were judged to have a critical overall risk of bias, mainly due to confounding. The absence of a concurrent control group was a major concern, as it limited the ability to distinguish intervention-related effects from changes occurring independently of the intervention, including temporal fluctuations in disease activity, regression to the mean, or other concomitant factors. Accordingly, the confounding domain was classified as critical in both studies. Selection of participants, deviations from the intended interventions, and reporting were consistently rated as presenting a moderate risk of bias, whereas classification of interventions and missing outcome data were judged to have a low risk of bias in both studies. Outcome measurements showed some variability, with one study classified as having a moderate risk and the other as having a serious risk of bias. The use of ROBINS-I was considered appropriate because the tool explicitly addresses the absence of a concurrent control group within the confounding domain and allows for a structured evaluation of time-related biases. However, given the critical overall risk of bias assigned to both studies, the observed within-study changes should be regarded as hypothesis-generating rather than confirmatory.

Among the randomized controlled trials, Biedermann et al. (2024) [40] was judged to have some concerns regarding the overall risk of bias. The domains of randomization, deviations from the intended interventions, and measurement of the outcome were rated as having a low risk of bias, whereas some concerns were identified for missing outcome data and selection of the reported result.

Secondary publications arising from overlapping patient cohorts were not considered independent clinical studies. Roth et al. (2016) [58] was treated as a secondary report of the Biedermann et al. (2013) [39] cohort, whereas Zobrist et al. (2024) [59] was treated as a secondary analysis of the randomized trial reported by Biedermann et al. (2024) [40]. Accordingly, these reports were not independently counted in the risk-of-bias assessment.

The overall distribution of risk-of-bias judgments across the ROBINS-I and RoB 2 domains is illustrated in Figure 4 and Figure 5, respectively.

## 4. Discussion

The present systematic review provides an overview of the available evidence on berry-derived and berry-based interventions in inflammatory bowel diseases, with particular attention to their phytochemical composition, polyphenol and/or anthocyanin content, and potential role in inflammatory gastrointestinal diseases. No temporal restrictions were applied to the literature search, allowing for a comprehensive assessment of the available evidence. Overall, the selected studies indicate a growing interest in berry-derived and berry-based interventions as potential strategies in IBD [60,61]. However, the evidence remains predominantly preclinical, while clinical studies are limited and characterized by substantial methodological and biological heterogeneity.

The qualitative analysis of preclinical studies revealed a predominant pattern of beneficial effects across different experimental models of intestinal inflammation. However, differences in animal models, berry species, interventions, dosages, and experimental conditions limit direct comparisons between studies.

In several murine models of DSS- or TNBS-induced colitis, supplementation with blueberry extract, blueberry anthocyanin fraction, fermented goji berry juice, maqui berry extract, and American cranberry was associated with reduced colonic levels of TNF-α, IL-6, and IL-1β compared with untreated controls [49,52,54,56], supporting the biological activity of berry-based interventions in experimental models of intestinal inflammation.

In particular, in the TNBS model, maqui berry extract reduced phosphorylation of p65, suggesting modulation of the NF-κB pathway [52]. These findings suggest that modulation of inflammatory signaling pathways may contribute to the biological effects of berry-derived interventions.

Additional preclinical evidence suggests that berry-based interventions may influence intestinal barrier integrity. In DSS-treated mice, Solanum nigrum juice preserved intestinal morphology and increased the expression of TJP1 and MKi67, markers associated with tight junction integrity and epithelial proliferation, respectively, as reported in Xing et al. [57]. These observations raise the possibility that the beneficial effects of berry-based interventions may involve not only attenuation of inflammatory signaling but also preservation of epithelial homeostasis.

The gut microbiota represents another potentially relevant mechanism linking berry-derived compounds to intestinal inflammation. In IL-10 knockout mice supplemented with grape seed extract, increased abundances of *Lactobacillus* and *Bacteroides* were associated with clinical improvement and reduced colonic oxidative stress [54], while cranberry supplementation in DSS-treated mice was associated with modulation of DSS-induced dysbiosis and reduced levels of pro-inflammatory cytokines [56].

An important characteristic of the preclinical evidence is the substantial heterogeneity of the interventions investigated. The included studies evaluated whole berries, fruit powders, juices, fermented preparations, extracts, anthocyanin-rich fractions, oils, and other berry-derived preparations. These preparations differed considerably in their phytochemical composition and polyphenol content, while doses and treatment durations varied substantially. Moreover, the experimental models also reproduced different aspects of intestinal inflammation, including acute chemically induced colitis and chronic or genetically driven inflammation. Consequently, although several studies reported similar biological outcomes, the evidence cannot be interpreted as demonstrating a uniform effect of berry-derived compositions across different preparations or disease contexts.

This distinction becomes relevant when considering the clinical evidence, which is considerably more limited than the preclinical evidence and is also characterized by substantial heterogeneity. Most clinical evidence concerned UC, whereas data in CD were scarce. Differences in berry species, formulation, dose, treatment duration, disease activity, concomitant therapy, and outcome assessment make direct comparisons difficult and limit the possibility of identifying a reproducible dose–response relationship.

Some clinical studies of bilberry supplementation reported reductions in fecal calprotectin and improvements in endoscopic and histological scores, without serious adverse events being reported [39,58].

These observations provide preliminary support for a potential anti-inflammatory effect in UC. However, a more recent multicenter randomized controlled trial of an anthocyanin-rich bilberry extract in patients with active moderate-to-severe UC showed that although fecal calprotectin was significantly reduced in the intervention group, the primary clinical outcome was not significantly superior to placebo [39].

Similarly, the microbiome analysis of participants from the same trial did not demonstrate significant overall changes in gut microbiota composition, although exploratory associations between specific microbial features and fecal calprotectin were observed [59]. These findings highlight the complexity of establishing a link between changes in individual biological markers or microbial features and clinical response.

Evidence in CD remains particularly limited. This may also reflect the substantial heterogeneity of the disease, including differences in disease location, phenotype, severity, and clinical manifestations, which may complicate the evaluation of dietary and nutraceutical interventions. The pilot study of freeze-dried grape powder did not demonstrate significant overall changes in the Harvey–Bradshaw Index, fecal myeloperoxidase, or hsCRP, although individual reductions in fecal MPO and modest changes in gut microbiota composition were observed [41].

These clinical findings suggest that selected berry-derived interventions may influence inflammatory and other biological markers. Several factors may explain the discrepancy between the relatively coherent preclinical evidence and the less conclusive clinical findings. Polyphenol metabolism is highly dependent on individual and microbial characteristics, and the conversion of compounds such as anthocyanins and ellagitannins into bioactive metabolites may vary according to gut microbiota composition [62,63].

This may be relevant in IBD, where disease-associated alterations in the gut microbiota could influence the production and bioavailability of these metabolites. This aspect is particularly relevant considering that the biological activity of ellagitannins may depend, at least in part, on their microbiota-mediated conversion into urolithins, which can accumulate in colonic tissues and have been reported to exert anti-inflammatory and antitumor effects [64].

Moreover, the formulations used in the included studies were not standardized. They ranged from whole fruits and freeze-dried powders to fermented juices and purified anthocyanin extracts. The polyphenol content was not consistently characterized using comparable measures, limiting the possibility of establishing meaningful dose–response relationships.

Differences in formulation, bioavailability, metabolism, and exposure to individual polyphenols and their metabolites may therefore contribute to the variability observed across studies.

The risk of bias assessment highlighted important methodological considerations between clinical and preclinical evidence. Preclinical studies showed a predominance of ‘unclear risk of bias’ judgments across most domains. Allocation concealment, random housing, and random outcome assessment were classified as unclear in all studies, while blinding of participants/personnel and outcome assessors was also insufficiently reported in most studies. Importantly, no domain was classified as high risk of bias in preclinical studies.

Clinical non-randomized studies were judged to have a critical overall risk of bias, mainly because of confounding and the absence of a concurrent control group. Therefore, the findings from these single-arm studies should be interpreted with caution, as the lack of a concurrent comparator limits causal inference. Changes observed between baseline and follow-up may reflect the natural fluctuation of inflammatory bowel disease, regression to the mean, concomitant treatments, or other factors occurring over time, rather than an effect of the intervention itself. Accordingly, these findings should be considered as preliminary evidence and should not be interpreted as definitive evidence of an intervention effect.

The randomized controlled trial was judged to have some concerns regarding the overall risk of bias, particularly regarding missing outcome data and selection of the reported results.

Importantly, the current evidence supports a biological rationale for berry-derived and berry-based interventions as potential adjunctive strategies in IBD, and particularly in UC. However, heterogeneity in the type of berry-derived intervention, formulation, dose, treatment duration, and outcomes assessed, together with the methodological limitations identified by the risk-of-bias assessment, prevents firm conclusions regarding therapeutic efficacy. Consequently, the findings cannot be considered sufficient to establish a general therapeutic effect of berry-derived and berry-based interventions.

The apparent consistency between preclinical mechanisms and the limited clinical observations should therefore be interpreted as evidence supporting biological plausibility rather than as confirmation of therapeutic efficacy. The translational gap between preclinical and clinical evidence may be influenced by several factors. While the preclinical evidence provides a possible biological rationale for the intervention, the current clinical evidence remains limited by methodological weaknesses. Further studies are warranted to evaluate whether the findings observed in preclinical models can be confirmed in humans and whether the biological effects of berry-derived and berry-based interventions may translate into clinically relevant benefits for patients with IBD. Overall, the preclinical findings and the limited clinical evidence currently available suggest that this field merits further investigation through well-designed clinical studies and standardized interventions, which may also include nutritional and rehabilitation approaches aimed at supporting patients’ functional status, physical activity, and quality of life.

## 5. Conclusions

In conclusion, the available evidence suggests that berry-derived and berry-based interventions may exert biological effects relevant to intestinal inflammation, as supported by findings from preclinical models and preliminary observations in patients with IBD, particularly UC. However, the limited number of clinical studies, together with the substantial heterogeneity in berry species, formulations, doses, treatment duration, and outcome measures, as well as the methodological limitations identified, precludes firm conclusions regarding their therapeutic efficacy. Therefore, current evidence should be interpreted as supporting biological plausibility rather than establishing a clinical benefit. Further well-designed clinical studies using standardized and well-characterized interventions are needed to clarify whether the biological effects observed in preclinical models can be consistently reproduced in patients with IBD.

## 6. Limits of Study

Several limitations should be considered when interpreting the findings of this systematic review.

The pathophysiology of IBD is highly complex and multifactorial, involving genetic susceptibility, immune dysregulation, gut microbiota alterations, epithelial barrier dysfunction, and environmental or dietary factors. As a result, isolating the effect of berry consumption or berry-derived interventions is challenging, and observed outcomes may be influenced by multiple confounders, including background diet, medication use, and lifestyle factors. Preclinical studies included in this review were characterized by heterogeneity in experimental design, with different animal species and models of colitis, including DSS, TNBS, and IL-10^−^/^−^ models. Each model reproduces distinct aspects of IBD pathophysiology, limiting the direct comparison of results across studies.

Clinical outcomes were heterogeneous and included fecal calprotectin, fecal myeloperoxidase, endoscopic and histological assessments, inflammatory biomarkers, and gut microbiota analyses. This variability further limited comparability across studies and precluded meaningful quantitative evidence synthesis.

Furthermore, few studies provided detailed characterization of the phytochemical composition and bioavailability of the tested formulations. Since the biological activity of berry-derived and berry-based interventions is strongly influenced by intestinal microbial metabolism and individual variability in gut microbiota composition, differences in polyphenol absorption and metabolite production may have contributed to the variability of the reported outcomes.

Potential sources of bias should be acknowledged. Studies reporting positive effects of berries on IBD may be more likely to be published, leading to publication bias. Some studies may not report all measured outcomes, resulting in incomplete evidence synthesis.

The clinical studies also presented important methodological limitations. The non-randomized studies were mainly affected by potential confounding related to the absence of a concurrent control group, while the randomized controlled trials showed either some concerns or a high overall risk of bias. These limitations reduce confidence in the available clinical evidence and should be considered when interpreting the reported effects.

In addition, limitations related to the databases searched, language restrictions, or availability of full-text publications may have resulted in the exclusion of potentially relevant studies.

Taken together, these limitations suggest that while the current evidence indicates potential benefits of berry-derived and berry-based interventions for IBD, the results should be interpreted with caution, and further high-quality, standardized studies are needed.

## Figures and Tables

**Figure 1:**
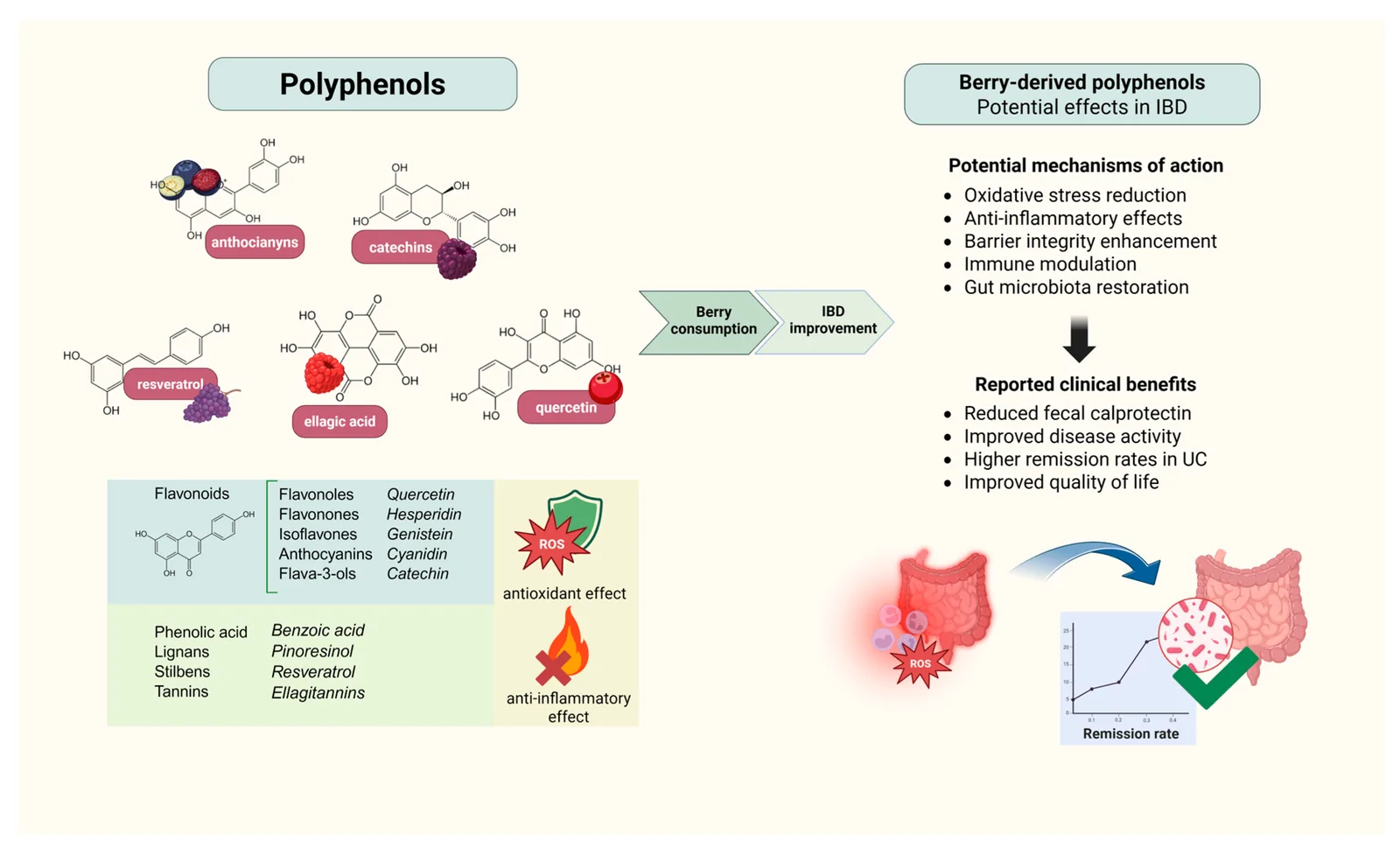
Polyphenol content and properties of berry-derived and berry-based interventions. ROS, Reactive Oxygen Species; IBD, Inflammatory Bowel Disease.

**Figure 2:**
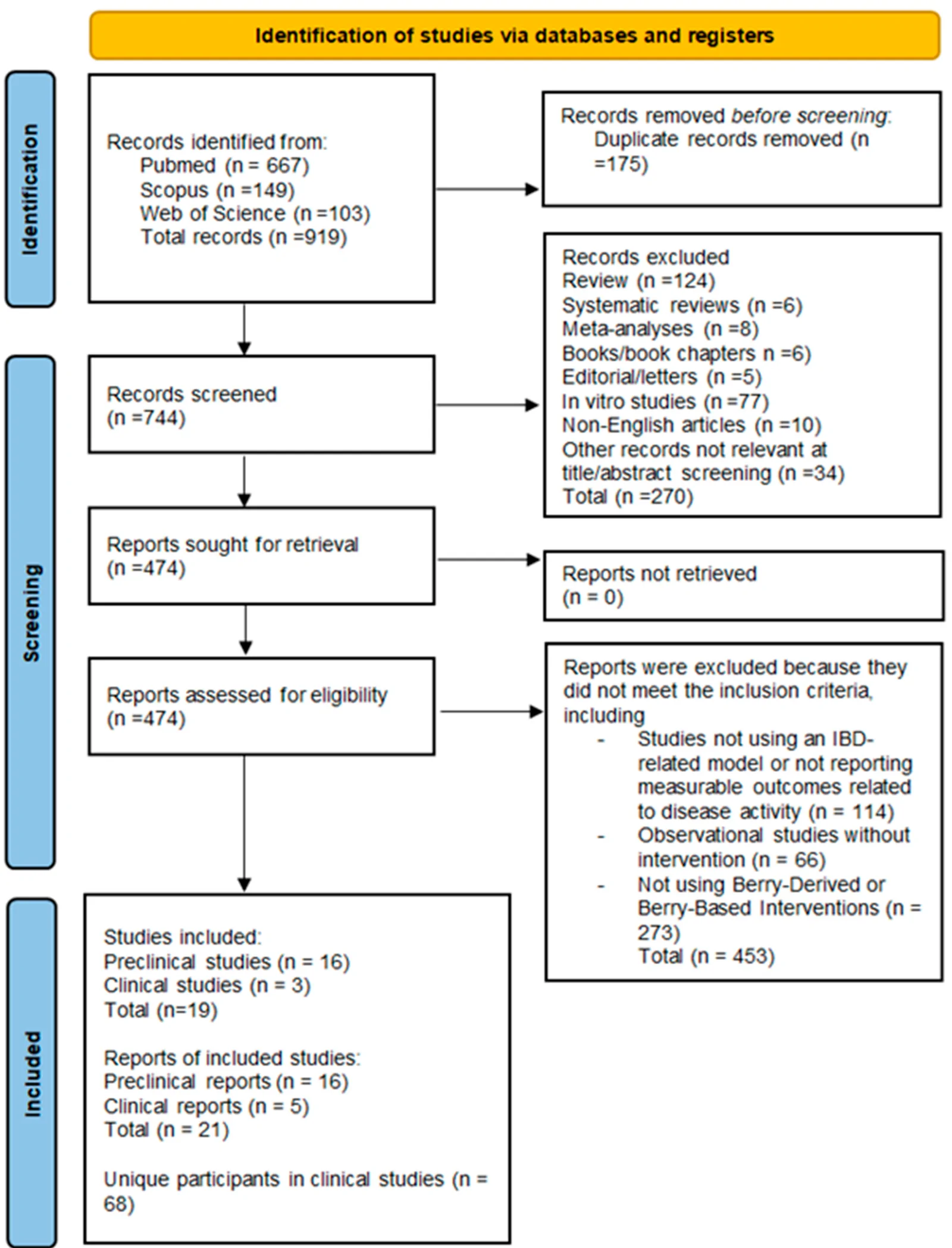
PRISMA Flow chart.

**Figure 3:**
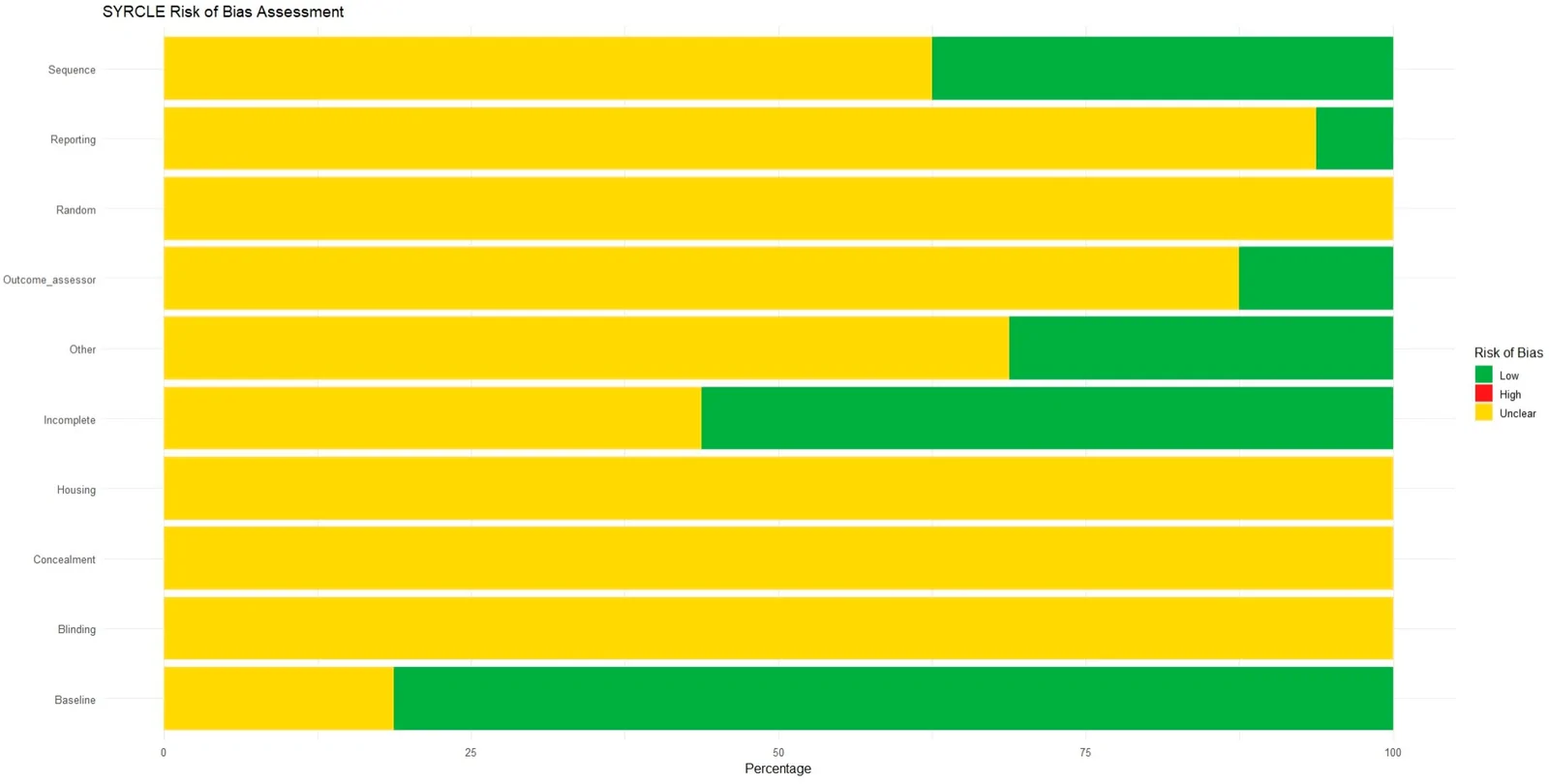
Risk of bias assessment of the included animal studies across the individual SYRCLE domains. The figure shows the distribution of judgments classified as low risk, unclear risk, or high risk of bias for each domain. High (red) is missing from the graph.

**Figure 4:**
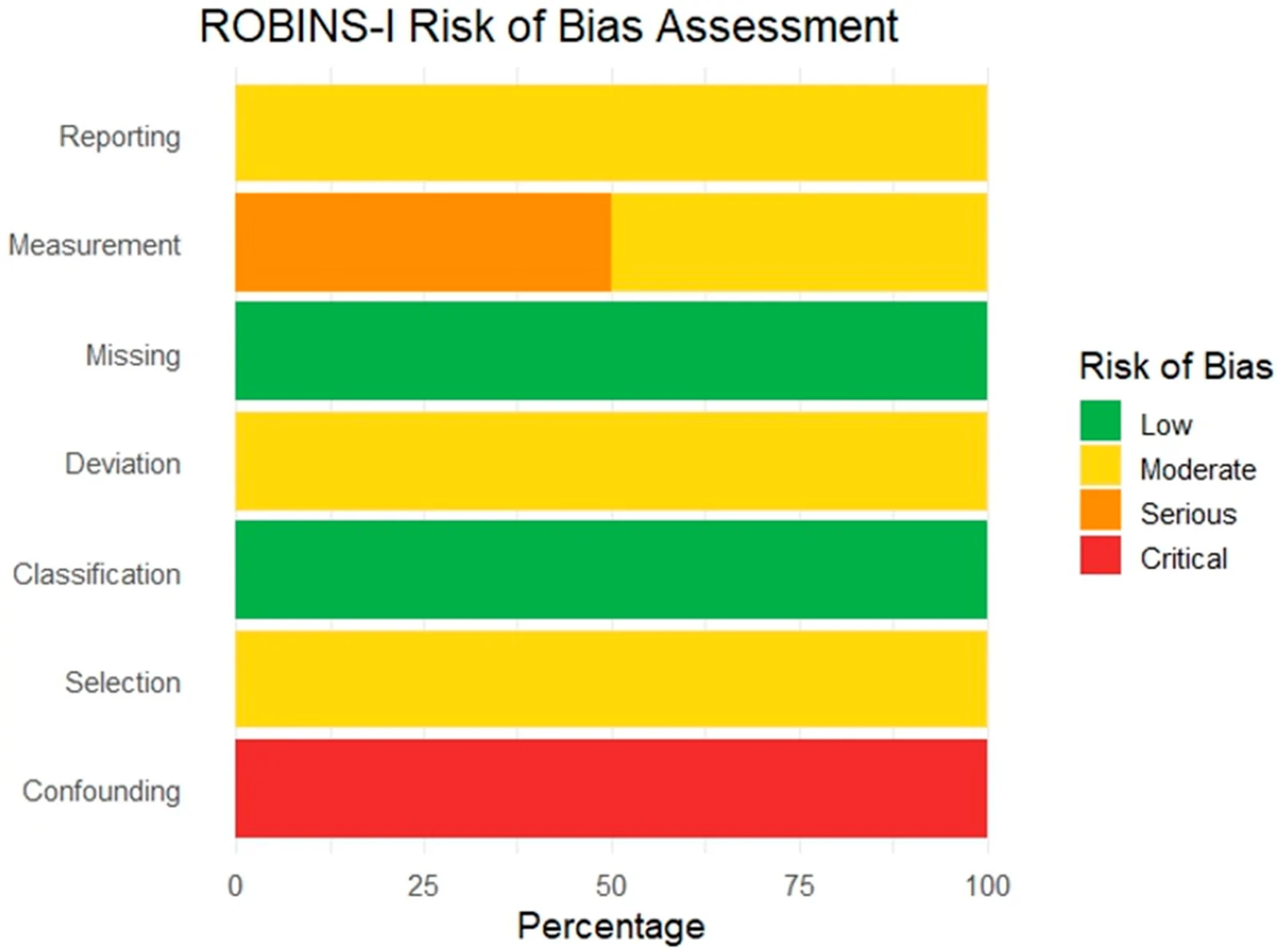
Risk of bias assessment of the non-randomized clinical study by using the ROBINS-I tool. The assessment covers the individual domains related to confounding, selection of participants, classification of interventions, deviations from intended interventions, missing data, measurement of outcomes, and selection of the reported result.

**Figure 5:**
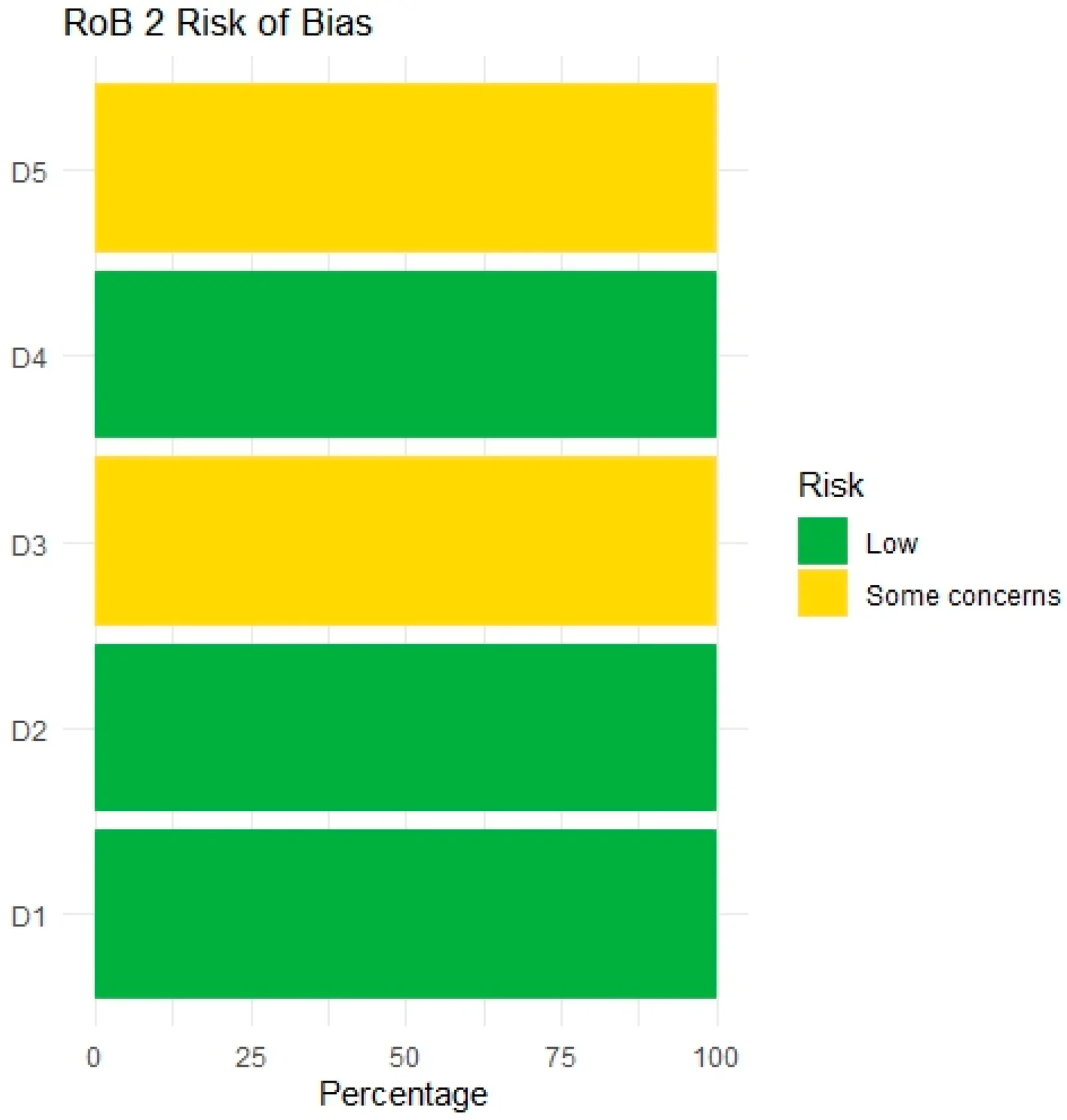
Risk of bias assessment of the randomized controlled trial using the Cochrane RoB 2 tool. The assessment covers the five domains: randomization process (D1), deviations from intended interventions (D2), missing outcome data (D3), measurement of the outcome (D4), and selection of the reported result (D5).

**Table 1:** PICOS framework for preclinical studies.

Element	Description
Population	Rodent models of experimental IBD (DSS, TNBS, IL-10^−^/^−^).
Intervention	Berry-derived and berry-based interventions, including whole berries, berry extracts, juices, powders, fermented products, anthocyanin-rich fractions, and other phytochemically characterized berry-derived preparations.
Comparison	Healthy animals or untreated colitis controls; conventional therapy when available.
Outcomes	Disease activity index, colon length, histology, cytokines, oxidative stress, gut microbiota, and intestinal barrier markers.
Study design	Original in vivo animal studies.

**Table 2:** PICOS framework for clinical studies.

Element	Description
Population	Adult patients with ulcerative colitis or Crohn’s disease.
Intervention	Berry-derived or berry-based interventions, including whole-fruit preparations, extracts, juices, powders, and standardized polyphenol- or anthocyanin-rich formulations.
Comparison	Placebo, standard care, or baseline values.
Outcomes	Clinical remission, Mayo score, fecal calprotectin, inflammatory biomarkers, quality of life, microbiota composition, adverse events.
Study design	Clinical trials, randomized controlled trials, and pilot studies.

**Table 3:** Preclinical evidence in IBD.

Animal Models	Size of Group (Number of Animals)	Sex	Type of Berry	Phytochemical Characterization	Treatment (Dose and Administration Route)	Duration	Comparator	Primary Outcome	Effect Estimate or Confidence Interval	Adverse Events	Results	Ref.
DSS-induced colitis in male C57BL mice	4 groups, (N not specified)	M	Blueberry extract	86 anthocyanin-related compounds (including cyanidins, delphinidins, malvidins, peonidins, petunidins) extracted by LC-MS spectroscopy	200 mg/kg/day by oral gavage (10 mL/Kg)	21 days	Control vehicle	Disease activity index (DAI) score	NR	NR	Protective effects of blueberry extract: mice showed significant improvements compared to controls, including reduced body weight loss and less shortening of the colon length; ER stress-mediated apoptosis-driven protective effects of blueberries in ameliorating DSS-induced IBD	[42]
DSS-induced colitis in Sprague-Dawley rats	36(6 groups, N = 6/group)	NR	Blueberry	NR	Freeze-dried blueberry powder, 5 g/rat/day, orally with the diet	17 days (7 days before + 10 days during DSS induction)	Colitis Control group	Disease activity index (DAI)	NR	NR	Blueberry alone significantly reduced Disease Activity Index (DAI), colonic MPO activity, MDA levels, bacterial translocation to the liver and mesenteric lymph nodes, and caecal *Enterobacteriaceae* counts compared with the colitis control.	[43]
DSS-induced colitis in Sprague-Dawley rats	36 (6 groups, N = 6/group)	F	Blueberry husks	NR	80 g dietary fibre/kg diet (equivalent to 196.1 g blueberry husks/kg diet), orally with the diet	14 days (7-day adaptation + 7-day experimental period)	Colitis control group	Disease activity index (DAI)	NR	NR	Blueberry husks attenuated DSS-induced colitis, reducing DAI, body-weight loss, MDA levels, serum IL-12, *Enterobacteriaceae* counts, and bacterial translocation, while increasing caecal SCFA, particularly acetate and butyrate.	[44]
TNBS-induced colitis in Wistar rat	40 (4 groups, N = 10/group)	M	Blueberry	Anthocyanin-rich fraction (ARF) contained 10.28 ± 0.48 g/L total anthocyanins, corresponding to 98.75 ± 5.71 mg/100g of fruit. Total phenolic content was 12.92 ± 1.59 g/L (119.96 ± 14.80 mg/100g of fruit). HPLC-DAD identified 15 anthocyanin peaks, mainly malvidin, petunidin, peonidin, delphinidin and cyanidin glycosides; malvidin-3-galactoside (20.93%) and petunidin-3-arabinoside (18.70%) were predominant compounds.	10 mg/kg/day by intragastric infusion	8 days	TNBS-induced colitis control group	Body weight variation and macroscopic colonic damage score	NR	NR	ARF more effectively reduced colonic damage, leukocyte infiltration, and oxidative stress. It significantly suppressed COX-2 and iNOS expression, while 5-ASA only affected COX-2. Superior ability of anthocyanins to downregulate iNOS expression and reduce leukocyte infiltration.	[45]
DSS-induced colitis model in C57BL6/J mice	18–21 (3 groups, N = 6–7/group)	M	Goji berry (Wolfberry, WOL)	NR	2% WOL powder incorporated into the AIN-93G diet	WOL 7 days before DSS exposure + 9 days of DSS administration	DSS control group	Disease activity index (DAI)	NR	NR	WOL supplementation significantly ameliorated DSS-induced colitis and improved intestinal mucosal barrier integrity. DAI decreased, colon shortening was attenuated, plasma MMP-3 levels were significantly reduced, and relative mesenteric fat weight was significantly improved. Transcriptomic/proteomic analyses indicated reduced inflammatory and extracellular-matrix-remodeling response	[46]
DSS-induced colitis in C57BL/6 mice	29 (2 groups, N = 15 control diet; N = 14 diet supplemented with Goji)	M	Goji berry	NR	1% *w*/*w* Goji powder incorporated into the AIN-93G diet	4 weeks of supplementation, followed by 7 days of DSS exposure and 7 days of recovery	DSS-induced colitis without Goji berry supplementation	Disease activity index (DAI)	NR	NR	Goji attenuated DSS-induced body-weight loss, diarrhea, fecal bleeding, disease activity index, colon shortening, and histological damage. Reduced neutrophil infiltration and IL-6, COX-2, CXCL1 and MCP-1 expression. No significant improvement was demonstrated for some tight junction/adhesion markers. Protective/preventive effect against DSS-induced colitis	[47]
IL-10-deficient spontaneous colitis mice	14 (2 groups, N = 7 control diet; N = 7 diet supplemented with Goji)	M	Goji berry	NR	1% *w*/*w* Goji powder	10 weeks	Standard rodent diet (Con), without Goji control group	Colonic pathobiological score	NR	NR	Goji improved colonic epithelial and crypt structure, reduced histopathological scores and macrophage infiltration, and decreased Il17a/Tgfb1 mRNA expression while increasing Muc1 expression and fecal IgA levels. Goji modulated the gut microbiota by increasing *Actinobacteria* and *Bifidobacteria* and promoting butyrate-producing bacteria	[48]
DSS-induced ulcerative colitis in C57BL/6 mice	40 (4 groups, N = 10/group)	M	Goji berry	NR	20 mL/kg/day of unfermented raw goji juice (UFRJ) or probiotic-fermented goji juice (FGJ), by gavage	30 days	Raw goji juice not fermented control group	Disease Activity Index (DAI)	NR	NR	Both unfermented and probiotic-fermented goji berry juice attenuated DSS-induced colitis, reduced oxidative stress, increased anti-inflammatory effects, and exerted beneficial effects on the gut microbiota, promoting the growth of beneficial bacterial populations	[49]
DSS-induced ulcerative colitis in FVB mice	20 (4 groups, N = 5/group)	M	Goji berry	Total flavonoid content, determined by the Dowd colorimetric method; *Lycium barbarum* lipid-derived nanoparticles (LBLNs) derived from the total fat-soluble fraction of *L. barbarum*	1 or 2 mg/kg/day, orally (p.o.)	5 days	DSS control group	Colitis severity (body weight loss, colon shortening, spleen weight and histopathological damage).	NR	NR	Treatment reduced body-weight loss, colon shortening, spleen enlargement, colonic MPO activity and TNF-α levels. Histological inflammation, crypt damage and ulceration were markedly reduced. LBLNs also restored the expression of the tight-junction proteins occludin and ZO-1, indicating improved intestinal barrier integrity	[50]
DSS-induced ulcerative colitis in C57BL/6 mice	60 (6 groups, N = 10/group)	M	Maqui berry	Total phenolics, flavonoids, anthocyanins, and proanthocyanidins were quantified in petroleum ether, chloroform, ethyl acetate, n-butanol, and residual water fractions of maqui berry extract	50, 100, or 200 mg/kg/day of Maqui berry water extract (MWE), ethyl acetate fraction, by intragastric administration	15 days	Control group	Inflammatory bowel disease index (IBD index)	NR	NR	MWE reduced DAI, colon shortening, and histopathological damage. It decreased colonic MDA and NO, increased GSH at 200 mg/kg, and reduced serum MPO and IL-1β at all doses and TNF-α at 200 mg/kg. Colonic iNOS and COX-2 expressions were reduced, while occludin expression was improved. MWE also partially corrected DSS-induced gut microbiota dysbiosis	[51]
TNBS-induced Crohn’s-like colitis in BALB/c mice	24 (4 groups, N = 6/group)	M	Maqui berry	Total polyphenolic content of 39.02 mg/g (expressed as gallic acid)	50 mg/kg/day of polyphenolic maqui extract (Ach), by gavage	Ach for 4 days after TNBS induction (Curative group); Ach for 7 days before and 4 days after TNBS induction (Preventive group)	Control group	Body weight changes, colon length, macroscopic colonic damage	NR	NR	Both preventive and curative Ach treatment attenuated body-weight loss and colon shortening, reduced macroscopic and microscopic colonic damage and transmural inflammation, improved mucosal architecture and mucus secretion, promoted M2 macrophage polarization, reduced COX-2 and iNOS expression, and modulated the Nrf2/HO-1 antioxidant pathway	[52]
TNBS-induced colitis in Wistar rats	41 (7 groups)	M	Grape	Total phenolics: 45.8 g GAE/kg; anthocyanins, quercetin (free and glucuronide), resveratrol, caffeoyltartaric, fertaric and caffeoylquinic acids, palmitic and linoleic acids identified by ESI(−)-MS	1% (1.18 g/kg/day) or 2% (2.36 g/kg/day) of G8000^TM^, by gavage, once daily	16 days	TNBS-induced colitis, untreated group	Immunoexpression of TNF-α, iNOS and COX-2, and peripheral blood DNA damage	NR	NR	Grape juice reduced TNF-α and iNOS immune expression at both 1% and 2% doses, while 1% grape juice also reduced COX-2 expression. Grape juice decreased peripheral blood DNA damage, particularly with 1% treatment administered 7 days and 2% treatment	[53]
IL-10-deficient spontaneous colitis mice	40 (4 groups)	M	Grape	Commercial grape seed extract (Gravinol-S), containing a minimum of ≥80% proanthocyanidins	1% of GSE (g GSE/g dry feed weight)	16 weeks	IL10KO-CON control group	Inflammatory bowel disease (IBD) indices, colitis severity	NR	NR	Dietary grape seed extract delayed and attenuated the development of IBD symptoms, increased colonic goblet cell density, reduced MPO mRNA and protein levels, attenuated colonic inflammation, decreased claudin-2 mRNA and protein expression, and increased the abundance of *Bacteroides* and *Lactobacilli* in the gut microbiota	[54]
DSS-induced colitis in BALB/c mice	44 (acute colitis); 48 (chronic colitis)	F	Bilberry	Anthocyanins (AC) (11.2% AC in dried bilberries; 60–70% anthocyanins in the AC extract)	20% dried blueberries or 1% and 10% AC extract incorporated into standard mouse chow	2 weeks pre-treatment + 7 days DSS (acute); 2 weeks pre-treatment + 49-day (chronic)	DSS-colitis control group	Disease severity and histological colitis score	NR	NR	Bilberry supplementation attenuated the severity of acute DSS-induced colitis, reducing the secretion of the pro-inflammatory cytokines TNF-α and IFN-γ from mesenteric lymph node cells. Dried bilberries also improved chronic DSS-induced colitis, while AC administration reduced intestinal inflammation in both acute and chronic colitis, as indicated by lower histological scores and cytokine secretion. Both bilberries and AC prevented inflammation-induced apoptosis in colonic epithelial cells	[55]
DSS-induced colitis in CD-1 mice	40 (4 groups, N = 10/group)	M	Cranberry	NR	Standard diet containing 1.5% (*w*/*w*) of freeze-dried whole cranberry powder in AIN-93G diet; (equivalent, for a 60 kg adult, to a daily dose of ≈7.5 g of whole cranberry powder or 65 g of fresh fruit)	37 days	DSS-treated mice receiving standard AIN-93G diet group	Disease Activity Index (DAI) and histological score colitis	NR	NR	Dietary whole cranberry significantly attenuated DSS-induced colitis, increasing colon length and reducing disease activity and histological scores, thereby alleviating colonic inflammation. Cranberry supplementation decreased colonic levels of the pro-inflammatory cytokines IL-1β, IL-6, and TNF-α. It also improved DSS-induced reductions in gut microbiota α-diversity and partially restored microbial composition, increasing potentially beneficial bacteria such as *Lactobacillus* and *Bifidobacterium* and decreasing potentially harmful bacteria such as *Sutterella* and *Bilophila*	[56]
DSS-induced ulcerative colitis in C57BL/6 N mice and Sprague-Dawley	3 groups for mice, 3 groups for rats	M	Nightshade (Berries of *Solanum nigrum* Linn.)	UPLC-Q-TOF-MS/MS and NMR identified 29 major components, including steroidal alkaloids and chlorogenic acid; major metabolites included (3β,12β,22α,25R)-3,12-dihydroxy-siprosol-5-en-27-oic acid, a reduction product of (12β,27)-dihydroxy-solasodine, and chlorogenic acid	0.1 mL/10g body weight/day of fresh nightshade juice (FNS) by gavage	14 days	DSS-treated mice without FNS	Colitis severity and intestinal tissue pathology	NR	NR	FNS improved survival, reduced body weight loss and clinical symptoms, delayed mortality, partially restored colon length, reduced intestinal necrosis, increased MKi67 and TJP1 expression, and preserved intestinal morphology and barrier integrity. FNS also modulated gut microbiota composition and regulated tight-junction proteins, proliferation-associated proteins, and pro-inflammatory cytokines. Eleven FNS compounds reduced ROS levels in polarized macrophages, while solaoiate showed anti-inflammatory activity by reducing IL-1β and TNF-α levels in vivo. FNS metabolites also reduced IL-1β levels	[57]

M = Male, F = Female, NR = Not Reported.

**Table 4:** Clinical evidence in IBD.

Type of Study	Number of Enrolled Patients and Relative Disease	Sex of Patients	Type of Berry	Phytochemical Characterization	Treatment	Duration of Treatment	Primary Outcome	Effect Estimate or Confidence Interval	Adverse Events	**Results**	**Ref.**
Open pilot trial	13 patients with mild to moderate UC	9 M, 4 F	Bilberry	Anthocyanin content was quantified by repetitive HPLC analysis, with a mean content of 210 mg per tray	160 g/day of standardized anthocyanin-rich bilberry preparation (≈95 g dry weight; ≈600 g fresh bilberries), providing ≈840 mg anthocyanins/day	6 weeks of treatment; total follow-up of 9 weeks	Remission at the end of treatment (CAI < 4).	N.R.	Dark-bluish to black discoloration of feces and tongue in all patients; slight tooth discoloration (n = 1); mild heartburn (n = 1); mild-to-moderate flatulence (33%); no serious adverse events.	After 6 weeks of bilberry treatment, 63.4% of patients achieved clinical remission and 90.9% showed a clinical response. The total Mayo score significantly decreased from 6.5 to 3.6, while fecal calprotectin levels decreased from a mean of 778 to 305 μg/g. The beneficial effect was further confirmed by reductions in endoscopic Mayo score and histological Riley index. After discontinuation of bilberry intake, calprotectin levels and disease activity increased again. No serious adverse events were reported	[39]
Multi-center, randomized, placebo-controlled, double blind study	34 with moderate-to-severe active UC (ACRE n = 24; placebo n = 10); Per-protocol analysis: ACRE n = 18; placebo n = 8	15 F/19 M; ACRE 10F/14M, placebo 5F/5M	Bilberry	Standardized bilberry extract containing ≥25% anthocyanidins, expressed as delphinidin equivalents (UV spectrophotometric assay)	Standardized anthocyanin-rich extract (ACRE)-based preparation (corresponding to 800–1000 mg of anthocyanins (AC)) administered orally, three times daily	8 weeks	Clinical response at week 8, defined as a reduction in total Mayo score (TMS) ≥ 3 points	OR: 1.667; 90% CI: 0.399–6.963	Stool discoloration; no serious adverse events directly attributed to the intervention	ACRE did not significantly improve clinical response at week 8 compared with placebo, and the primary endpoint was not met (9/18 vs. 3/8; *p* = 0.278). However, fecal calprotectin significantly decreased in the ACRE group from 1049 ± 1139 to 557 ± 756 μg/g (*p* = 0.035), whereas no significant change was observed with placebo. No significant safety concerns were identified, and the two serious adverse events were considered unrelated to the study treatment	[40]
Pilot study	21 patients with CD	11 F, 10 M	Grape	Catechins (Catechin 14.6 mg/kg +/− 0.718; Epicatechin 2.43 mg/kg +/− 0.12); Anthocyanins (Peonidin 2.62 mg/kg +/− 0.152; Cyanidin 0.24 mg/kg +/− 0.013; Malvidin 6.96 +/− 0.298) Flavonols (Kaempferol 0.139 mg/kg +/− 0.018; Isorhamnetin 0.145 mg/kg +/− 0.016) Stilbenes (Resveratrol 0.33 mg/kg +/− 0.061) Total Polyphenols (in gallic acid equivalents) 354 mg/100g	45 g/day of freeze-dried grape powder, oral dietary supplementation (equivalent to ≈1.5 cups of fresh grapes) consumed as 22.5 g packets twice daily	3 weeks	Fecal microbiota composition (16S rRNA sequencing): baseline (pre-intervention) and post-intervention.	N.R.	One participant reported abdominal pain and nausea following GP consumption; these symptoms resolved within three hours of ingestion. No serious adverse events were reported.	After 21 days of freeze-dried grape powder supplementation, 13/21 participants (61.9%) showed a reduction in fecal MPO, while 17/21 (80.9%) experienced either a reduction or no change in the HBI score. Microbiome analysis showed modest directional shifts, including enrichment of *Akkermansiaceae, Bacteroidaceae, Tannerellaceae, Rikenellaceae*, and *Monoglobaceae*. Although overall microbial community structure did not significantly change at the cohort level, individualized microbiome responses and functional pathway shifts were observed. Grape powder supplementation was safe and well tolerated	[41]

M = Male, F = Female, OR = odds ratio, 90% CI = 90% confidence interval (CI).

## Data Availability

No data was used for the research described in the article.

## Abbreviations

The following abbreviations are used in this manuscript:

BAC	Bioactive compounds
IBD	Inflammatory bowel disease
CD	Crohn’s disease
UC	Ulcerative colitis
TNF-α	Tumor necrosis factor-alpha
IL-1β	Interleukin-1 beta
IL-6	Interleukin-6
NF-κB	Nuclear factor kappa B
ROS	Reactive oxygen species
RNS	Reactive nitrogen species
SOD	Superoxide dismutase
GPx	Glutathione peroxidase
PLA2	Phospholipase A2
COX	Cyclooxygenase
LOX	Lipoxygenase
EGFRs	Epidermal growth factor receptors
EAC	Ehrlich ascites carcinoma
AgNORs	Argyrophilic nucleolar organizer regions
EGCG	Epigallocatechin-3-gallate
Foxp3	Forkhead Box P3
IL-10	Interleukin-10
PAL	Phenylalanine ammonia-lyase
IMIDs	immune-mediated inflammatory diseases
CRP	C-reactive protein
ESR	Erythrocyte sedimentation rate
MAPK	Mitogen-activated protein kinase
ERK	Extracellular signal-regulated kinase
NOS	Nitric oxide synthase
COX-2	cyclooxygenase-2
LPS	Lipopolysaccharide
STAT	Signal transducer and transcription activator
IFN)-α	Interferon-α
GM-CSF	Granulocyte-macrophage colony-stimulating factor
EPO	Erythropoietin
TYK2	Tyrosine Kinase 2
6-MP	6-Mercaptopurine
RSV	Resveratrol
ET	Ellagitannin
PRISMA	Preferred Reporting Items for Systematic Reviews and Meta-Analyses
PICOs	Population Intervention Comparison Outcome Study design
ARF	Anthocyanin-rich fraction
5-ASA	5-aminosalicylic acid
TNBS	Trinitro benzenesulfonic acid
DSS	Dextran sodium sulfate
DAI	Disease activity index
ARBE	Anthocyanin-rich bilberry extract
MCP-1	Monocyte chemoattractant protein-1
SCFAs	Short-chain fatty acids
